# Autologous Bone Grafting in Trauma and Orthopaedic Surgery: An Evidence-Based Narrative Review

**DOI:** 10.3390/jcm10194347

**Published:** 2021-09-24

**Authors:** Filippo Migliorini, Francesco Cuozzo, Ernesto Torsiello, Filippo Spiezia, Francesco Oliva, Nicola Maffulli

**Affiliations:** 1 Department of Orthopaedics, University Clinic Aachen, RWTH Aachen University Clinic, 52074 Aachen, Germany; 2 Department of Medicine, Surgery and Dentistry, University of Salerno, Via S. Allende, 84081 Salerno, Italy; fra.cuoz@gmail.com (F.C.); ernesto.torsiello94@gmail.com (E.T.); olivafrancesco@hotmail.com (F.O.); n.maffulli@qmul.ac.uk (N.M.); 3 Ospedale San Carlo Potenza, Via Potito Petrone, 85100 Potenza, Italy; spieziafilippo@gmail.com; 4 School of Pharmacy and Bioengineering, Keele University Faculty of Medicine, Thornburrow Drive, Stoke on Trent ST4 7QB, UK; 5 Centre for Sports and Exercise Medicine, Barts and the London School of Medicine and Dentistry, Mile End Hospital, London E1 4DG, UK

**Keywords:** autologous, bone, graft, iliac crest, Reamer Irrigator Aspirator

## Abstract

Autologous bone grafting is common in trauma and orthopaedic surgery. Both the Reamer Irrigator Aspirator (RIA) and Iliac Crest Bone Graft (ICBG) aim to obtain autologous bone graft. Although the process of harvesting a bone graft is considered simple, complications may occur. This study examined morbidity and pain at the donor site, blood loss, and iatrogenic fractures, comparing RIA and ICBG. The source of the autologous bone graft, the alternative graft sites, and the storage modalities of the harvested bone marrow were also evaluated. In May 2021, PubMed, Embase, Scopus, and Google Scholar were accessed, with no time constraints. RIA may produce greater blood loss, but with less morbidity and complications, making it a potential alternative source of bone grafting.

## 1. Introduction

**Figure 1:**
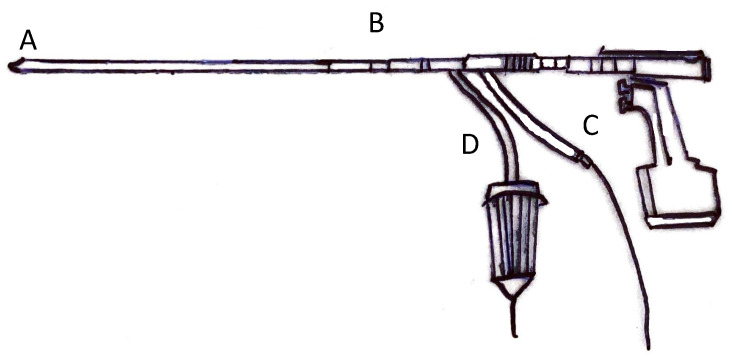
RIA device (A: portal to collect reaming; B: reamer driver shaft; C: water irrigator; D: aspirator/filtered canister).

Autologous bone grafting in trauma and orthopaedic surgery is common [1]. The most common indications for grafting are long bone non-union and arthrodesis, followed by osteomyelitis and maxillofacial operations [2,3]. The Iliac Crest Bone Graft (ICBG) was long considered the gold standard harvesting site [3]. Within the iliac crest, given its easier surgical access, the anterior portion is the most used harvest site [4]. However, though of simple execution, anterior ICBG may expose patients to a greater rate of side effects compared to the posterior ICBG [5]. In the past decade, the Reamer Irrigator Aspirator (RIA) has been introduced (Figure 1). RIA is supposed to be less invasive, allowing the harvest of greater graft volume directly from the medullary canal of long bones [6]. Bone grafting is common, and complications are often underestimated [7]. Discomfort and difficulties in sexual or daily activities may occur following ICBG [3]. The mean bone marrow volume harvested following ICBG is usually about 25 cc, which is considerably less compared to the RIA, whose harvesting volume is around 40 cc [8]. Although RIA is less invasive and exposes patients to less complication than ICBG, it may produce greater blood loss [9]. Moreover, RIA requires special instruments availability, fluoroscopy, and patient installation [10]. Patients are positioned supine, after anaesthesia. After locating the piriformis fossa using a percutaneous technique with the aid of biplanar fluoroscopy, a drill bit is used to open the entry site. Finally, the RIA is inserted to harvest bone graft. This includes attaching an appropriately sized reamer head, a saline bag to the irrigation port of three or five litres, an aspiration port, and a screen trap in line with suction tubing. Gravity flow and vacuum suction are used to maintain irrigation flow [11].

The present study clarifies pros and cons of RIA and ICBG as sources of autologous bone graft, discussing indications, bone graft volumes, complications, and the alternative grafting sites. Moreover, donor site morbidity, post-operative pain, and estimated blood loss between the two techniques (ICBG and RIA) are also discussed. 

## 2. Indications and Surgical Technique

### 2.1. Sources of Autologous Bone Grafts

Autologous cortical, cancellous, cortico-cancellous, and vascularized bone grafts can be used [12,13]. Cancellous bone is rich in mesenchymal stem cells with high osteogenic and osteoconductive potential [7,14,15]. Its trabecular structure allows rapid revascularization, usually within 48 h after transplantation [12,16,17]. The new bone formation is already demonstrable a few weeks postoperatively, and remodelling occurs within two months, with complete graft osteointegration achieved after approximately one year [12,18]. Given its proprieties, cancellous bone graft is employed to enhance fracture healing and arthrodesis, and to fill bone defects [19,20,21,22]. Cortical bone graft shows moderate osteoconductive, osteoinductive, and osteogenic capabilities, together with adequate mechanical stability [7,23,24]. However, given its dense cortical matrix, revascularization can take up to two months [7,25]. Cortical grafts are less used because of their lower osteoconductive and osteogenic proprieties, and they are mostly employed when it is necessary to repair segmental bone defects smaller than 5 cm [23]. Corticocancellous bone grafts offer the advantages of both cortical and cancellous bone [7,15]. 

Vascularized bone grafts, another valuable option [26,27], are usually harvested with a vascular pedicle, which is supposed to accelerate graft incorporation [25,26,28]. The autologous iliac crest is considered the best source of non-vascularized tricortical grafting, while the fibula is the best choice for mono- or bicortical vascularized grafts [29,30,31]. 

### 2.2. Indications

Bone grafting procedures span several specialties [32,33] and are especially used in orthopaedic, trauma, and reconstructive surgery [7,34,35]. Bone grafting is employed in defects resulting from fractures, non-union, tumours, and osteomyelitis [3,36]. Among them, fracture and long bone non-unions are the most common indications [3,11]. Cancellous bone grafting is commonly employed in sports medicine to repair subchondral bone defects deriving from trauma or to fill larger defects in patients with osteochondrosis dissecans [37,38,39]. Bone grafting combined with core decompression demonstrated satisfying outcomes in patients with osteonecrosis of the femoral head [40,41]. Posterior iliac crest bone grafting is commonly used in spine surgery to enhance arthrodesis [17,42,43,44,45,46]. 

### 2.3. Harvesting Technique

#### 2.3.1. ICBG Harvest

**Figure 2:**
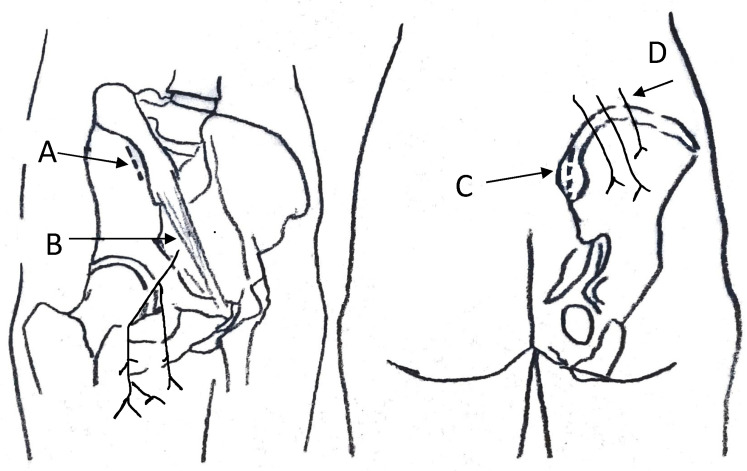
ICBG harvest site (A: anterior incision on anterior superior iliac spine; B: lateral femoral cutaneous nerve; C: posterior incision on posterior superior iliac spine; D: superior cluneal nerves).

Both the anterior and posterior iliac crest are sources of autograft (Figure 2) [47,48]. Usually, between 15 and 25 cc is harvested from the anterior and posterior iliac crest, respectively [5,49]. If more bone is required, anterior and posterior grafts can be harvested at the same surgical session [5]. Anterior iliac crest bone grafting is a technique of simple execution [50]. To avoid the lateral femoral cutaneous nerve, autografts are harvested from the gluteal tubercle using a skin incision parallel to the iliac crest, three centimetres posterior to the anterior iliac spine [33,51]. Electrocautery is used subperiosteally to elevate the external oblique muscles, avoiding the ilioinguinal and ilio-hypogastric nerves. The iliacus muscle can be lifted from the inner ileum table when greater exposure is required (cortico-cancellous or acetabular reamer harvest). Careful dissection, while preserving the anatomical planes, facilitates tissue closure at the end of the procedure [7]. In the posterior side harvesting, the skin incision is parallel to the iliac crest, and the posterior superior iliac spine is exposed subperiosteally, with the periosteum and dorsal-lumbar fascia preserved on the medial edge of the crest [33]. 

Alternative techniques are also available [52,53,54]. The trapdoor technique, the iliac crest-splitting technique, the trephine technique, the segmental bicortical or tricortical technique, the iliac crest aspirate, and the acetabular reamer technique have been described [49,52,53,54,55,56]. 

#### 2.3.2. RIA Harvest

**Figure 3:**
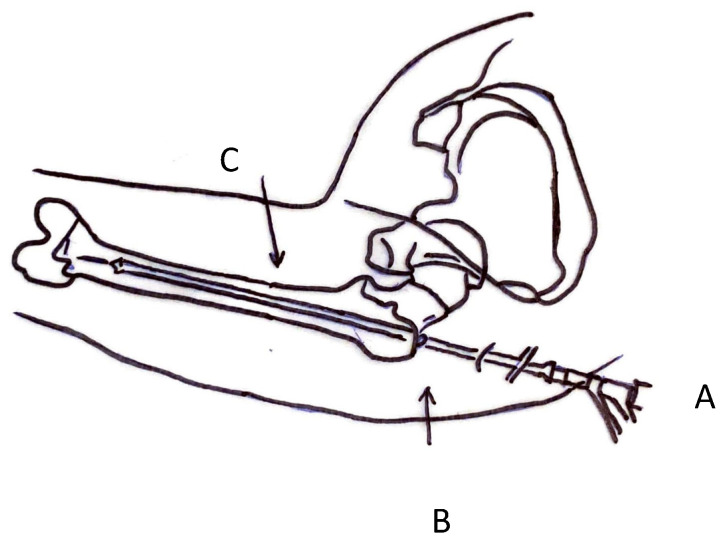
RIA access site (A: RIA inserted at trochanteric tip; B: level of great trochanter; C: anatomic axis of femur).

RIA system allows to harvest large amounts of autologous bone graft from the medullary canal of long bones (Figure 3) [57]. This device allows intramedullary reaming with simultaneous irrigation and aspiration [11]. 

The graft is harvested introducing the RIA into the medullary canal of the femur; by aspiration, the graft is collected in a filtered canister [57]. The femur is approached antegrade from the greater trochanter; since it causes less morbidity, this location is a valid alternative to the piriform approach [57]. Lateral and anterior–posterior radiographs allow to exactly position the RIA device. The RIA technique is versatile and has a short learning curve [57].

## 3. Storage and Alternative Grafting Sites

### 3.1. Storage, Management and Histological Profiles of Autologous Bone Graft

Storage of autologous bone is controversial [58]. The graft is normally stored in 0.9% saline solution or in a 5% glucose solution [59,60,61]. Dry condition storage impairs cell metabolism [62]. Perioperative antibiotic therapy is mandatory to prevent early infections. Fresh autografts should receive 1 g of dry antibiotic powder [61]. Such use can increase local therapeutic levels for up to three weeks without systemic toxicity [63]. A randomized controlled trial in 96 patients with infected tibial non-union, followed for a mean of 4.5 years, showed a significantly greater reduction in the rate of infection in patients treated with organism-specific antibiotic-impregnated autologous cancellous bone graft. In this study, infection was cleared in 44 (96%) of 46 patients treated with antibiotic-impregnated bone graft compared with 41 (82%) of 50 treated with graft alone [7]. The histological profile also plays an important role for bone grafting. Sagi et al. compared the histological and molecular profiles of bone grafts from the most used techniques [16]. Macroscopically, the two graft materials present different physical characteristics [64]. The RIA graft material consists of very small bone fragments mixed with blood clots, and the general handling characteristics were similar to those of a fluid or semisolid. Microscopically, RIA graft contains many hematopoietic cells and a mix of small cortical and cancellous bone fragments, haversian elements, and intact vascular tissue [16]. In contrast, ICBG material tends to be well formed, with integrated fragments of cancellous bone, and its handling characteristics are those of a solid [16]. 

### 3.2. Alternative Grafting Sites

Given its easier and routinely accessible site, the iliac crest remains the preferred harvest site. Several alternative grafting sites to the iliac crest are available. The choice of the harvest site depends on the proximity to the surgical site. The proximal part of the tibia is easily accessible and rich in cancellous and corticocancellous bone [65,66,67]. Usually, about 25 cc of graft is harvested from the proximal tibia; nevertheless, in young adults with good bone stock up to 70 cc is available for harvesting [34,53,68,69]. The complication rate at the donor site is less than 2%, while haematomas have been reported in 15% of patients [67,68,70]. Less than 2% of patients reported long-term pain [71]. The distal part of the tibia is an alternative grafting site, particularly convenient in foot and ankle surgery, as it is adjacent to the operative field; it produces relatively little blood loss, and it is an easy source of small volumes of cancellous bone [18]. Furthermore, the technique of harvesting is simple and is associated with a low rate of infections and fragility fractures [20,38,72]. The calcaneus is another grafting site often used in foot and ankle surgery for osteoinductive purposes [73]. The greater trochanter is another useful source of bone grafting reserved for ipsilateral multifragmentary proximal femoral fractures [74,75]. Similarly, the distal end of the radius can provide almost 3 cc of cancellous graft for hand and upper limb surgery [72,76,77,78]. Regarding RIA, literature reports the tibia as an alternative grafting site, but currently it is not commonly used [79]. 

## 4. Complications

### 4.1. Donor Site Morbidity

#### 4.1.1. ICBG 

The RIA technique appears to be safe and well tolerated, while ICBG seems to be associated with greater incidence of morbidities [71,80,81]. Up to 8.6% of patients experienced donor site complications following ICBG; chronic pain represents the most common cause of complain, following by lateral femoral cutaneous nerve lesions and iliac wing fractures, which are far less common [82,83,84,85]. Complications can be major or minor [6]. Major complications occur when patients require additional hospital care related to graft site morbidity (intravenous therapy or debridement) [35,84]. Minor complications can be managed at home, for example, using oral antibiotics therapy [86]. Similarly, iliac bone crest pain or discomfort have been considered minor complications if pain is adequately controlled without opioid analgesics [87]. Another reported complication is impaired walking because of pain at the harvest site. In a previous report, 11 of 87 patients reported difficulties in the first six months after surgery [87]. Arrington et al. [88] reported a 10% rate of minor complications (e.g., superficial infections, seromas, minor hematomas) and a rate of 5.8% of major complications requiring a change of management, additional surgery, or a prolonged hospital stay. Banwart et al. [89] reported that, of 180 patients treated with ICBG, 10% experienced major complications (3 acute and 15 chronic), and 39% developed minor complications. Schnee et al. [41] conducted a study on 184 anterior ICBG harvests in 144 anterior cervical fusion procedures (114 anterior cervical discectomy and fusion, 30 corpectomy) with a mean follow-up of eight months. They reported that 2.8% of the patients underwent a second surgery at the donor site, 5.6% had minor infection or wound dehiscence, and 97% were satisfied with the wound appearance [41]. Furthermore, there was a substantial negative effect on the quality of life in patients who received anterior iliac crest harvest [41,90]. Patients had difficulty in dressing, carrying out household chores, walking, restrictions on work or daily life, and impaired sexual activity [41]. Wound cosmetic was also analysed. De Palma et al. [91] reported discomfort lasting more than one year in 36% of patients who had anterior ICBG harvested. Almost all of these authors agree that discomfort and pain following iliac crest bone harvest decreases over time. In contrast, Canady et al. [92] reported that in 50 ACBGs used for maxillofacial procedures no patients suffered from pain at the iliac crest donor site. However, this result could be related to the amount of bone graft harvested, which is significantly smaller in maxillofacial procedures. Pain is considered the most common complaint. In a previous study, of 87 patients treated with ICBG, 37 reported pain six months after surgery [17]. Patients typically describe their pain as sharp, tender, and penetrating [17]. Anterior autologous bone grafting has been associated with greater rate of pain [93]. Some authors reported limited physical activity, secondary to pain, especially during the immediate post-operative period [94]. Belthur et al. [57] also reported acute anterior iliac crest pain using a visual analogue scale for frequency and intensity, for a maximum total pain score of 20 points. Chronic pain lasting more than three months was reported by a small percentage of patients following anterior autologous bone grafting [83,95,96]. Only 2.8% of patients complained of persistent pain for more than three months after surgery [41]. Blood loss is another common complication [71]. ICBG is characterized by a low amount of blood loss [3]. Intraoperative blood loss from posterior iliac crest harvest was estimated at 75 mL [5,97]. Scharfenberger and Weber [98] evaluated the haemoglobin and haematocrit drop after intramedullary harvest in 11 patients. The mean drop in haemoglobin was 4.3 g/dL, and the mean drop in haematocrit was 11% [98].

#### 4.1.2. RIA 

RIA carries a lower complication rate [99]. No superficial or deep hematoma, infection or adipose embolism have been documented following the use of RIA (Table 1) [100]. In addition, lower overall pain scores have been reported in patients undergoing femoral RIA harvesting [32,101]. According to Stafford et al. [102], RIA produced no intraoperative or postoperative complications. Kanakaris et al., in a retrospective study on 18 patients, reported three complications: haematomas in two patients (11%), and persistent non-union in one patient [103]. Qvich et al. [2] assessed the donor site morbidity and the complication rate associated with the RIA; the complication rate in 204 RIA procedures in 184 patients was less than 2%.

While pain at the harvest site is the most common complication following ICBG, during RIA technique, which involves continuous aspiration and reaming, a large volume of blood could be accidentally aspirated [80]. Although the average blood loss is around 200 mL [107], higher blood losses have been reported after using the RIA device (Table 1) [3,10]. Unfortunately, quantifying the effective blood loss after the RIA procedure is complex, and future studies are required.

Iatrogenic fractures are rare (Table 1) [108]. The estimated rate of fractures was 0.9% for ICBG, and 1% following RIA [106,108], and it is strongly influenced by the local bone mineral density [109].

## 5. Conclusions

ICBG is a well-established and relatively simple technique that provides a good quantity/quality ratio of obtainable bone. On the other hand, it may predispose patients to complications. RIA is versatile and has a short learning curve, with low incidence of complications and little discomfort at the donor site. However, RIA has been associated with greater amount of blood loss, and its use is subject to availability of the necessary hardware. Further comparative investigations are required to establish the best strategy to obtain autologous bone graft.

## Figures and Tables

**Table 1:** Main morbidities after using RIA and ICBG.

Author et al. Year	Patients	Treatment	Acute Complications	Chronic Complications	Pain	Fractures	Infections	Haematoma/Seroma
Almaiman et al., 2013 [5]	372	Icbg			372	3		1
Banwart et al., 1995 [89]	180	Icbg	14	62				
Belthur et al., 2008 [57]	41	Ria				0	0	0
		Icbg					3	1
Beirne et al., 1996 [4]	137	Icbg					5	
Burstein et al., 2000 [104]	55	Icbg					1	1
Calori et al., 2014 [3]	35	Ria			0	0	0	
	35	Icbg			5	1	5	
Dawson et al., 2014 [8]	56	Ria			1	1	5	
	57	Icbg			3		4	
Delawi et al., 2007 [105]	71	Icbg						
Deorio et al., 2005 [94]	134	Icbg		10	108			
Fernyhough et al., 1992 [83]	147	Icbg		42	21			
Finkemeir et al., 2010 [10]	23	Ria					2	
Goulet et al., 1997 [17]	170	Icbg			28		4	
Han et al., 2015 [80]	57	Ria				1		
Haubruck et al., 2018 [81]	306	Ria				3		
Kanakaris et al., 2011 [103]	42	Ria						2
Merrit et al., 2010 [42]	92	Icbg					1	1
Metsemakers et al., 2019 [106]	72	Ria				2	3	
Pollock et al., 2008 [43]	77	Icbg		2	75			
Qvick et al., 2013 [2]	204	Ria						
Robertson et al., 2001 [44]	106	Icbg			13		1	
Schizas et al., 2009 [96]	59	Icbg	2		6			1
Schwartz et al., 2009 [45]	170	Icbg		3	13			1
Silber et al., 2003 [82]	134	Icbg					20	
Westrich et al., 2001 [56]	390	Ria			21		1	1

## Data Availability

Not applicable.

## References

[1] Sen M.K., Miclau T. (2007). Autologous iliac crest bone graft: Should it still be the gold standard for treating nonunions?. Injury.

[2] Qvick L.M., Ritter C.A., Mutty C.E., Rohrbacher B.J., Buyea C.M., Anders M.J. (2013). Donor site morbidity with reamer-irrigator-aspirator (RIA) use for autogenous bone graft harvesting in a single centre 204 case series. Injury.

[3] Calori G.M., Colombo M., Mazza E.L., Mazzola S., Malagoli E., Mineo G.V. (2014). Incidence of donor site morbidity following harvesting from iliac crest or RIA graft. Injury.

[4] Beirne J.C., Barry H.J., Brady F.A., Morris V.B. (1996). Donor site morbidity of the anterior iliac crest following cancellous bone harvest. Int. J. Oral Maxillofac. Surg..

[5] Ahlmann E., Patzakis M., Roidis N., Shepherd L., Holtom P. (2002). Comparison of anterior and posterior iliac crest bone grafts in terms of harvest-site morbidity and functional outcomes. J. Bone Jt. Surg. Am..

[6] Dimitriou R., Mataliotakis G.I., Angoules A.G., Kanakaris N.K., Giannoudis P.V. (2011). Complications following autologous bone graft harvesting from the iliac crest and using the RIA: A systematic review. Injury.

[7] Myeroff C., Archdeacon M. (2011). Autogenous bone graft: Donor sites and techniques. J. Bone Jt. Surg. Am..

[8] Dawson J., Kiner D., Gardner W., Swafford R., Nowotarski P.J. (2014). The reamer-irrigator-aspirator as a device for harvesting bone graft compared with iliac crest bone graft: Union rates and complications. J. Orthop. Trauma.

[9] Marchand L.S., Rothberg D.L., Kubiak E.N., Higgins T.F. (2017). Is This Autograft Worth It?: The Blood Loss and Transfusion Rates Associated with Reamer Irrigator Aspirator Bone Graft Harvest. J. Orthop. Trauma.

[10] Finkemeier C.G., Neiman R., Hallare D. (2010). RIA: One community’s experience. Orthop. Clin. N. Am..

[11] McCall T.A., Brokaw D.S., Jelen B.A., Scheid D.K., Scharfenberger A.V., Maar D.C., Green J.M., Shipps M.R., Stone M.B., Musapatika D. (2010). Treatment of large segmental bone defects with reamer-irrigator-aspirator bone graft: Technique and case series. Orthop. Clin. N. Am..

[12] Bauer T.W., Muschler G.F. (2000). Bone graft materials. An overview of the basic science. Clin. Orthop. Relat. Res..

[13] Burchardt H. (1987). Biology of bone transplantation. Orthop. Clin. N. Am..

[14] Khan S.N., Cammisa F.P., Sandhu H.S., Diwan A.D., Girardi F.P., Lane J.M. (2005). The biology of bone grafting. J. Am. Acad. Orthop. Surg..

[15] Baldwin P., Li D.J., Auston D.A., Mir H.S., Yoon R.S., Koval K.J. (2019). Autograft, Allograft, and Bone Graft Substitutes: Clinical Evidence and Indications for Use in the Setting of Orthopaedic Trauma Surgery. J. Orthop. Trauma.

[16] Sagi H.C., Young M.L., Gerstenfeld L., Einhorn T.A., Tornetta P. (2012). Qualitative and quantitative differences between bone graft obtained from the medullary canal (with a Reamer/Irrigator/Aspirator) and the iliac crest of the same patient. J. Bone Jt. Surg. Am..

[17] Goulet J.A., Senunas L.E., DeSilva G.L., Greenfield M.L. (1997). Autogenous iliac crest bone graft. Complications and functional assessment. Clin. Orthop. Relat. Res..

[18] Saltrick K.R., Caron M., Grossman J. (1996). Utilization of autogenous corticocancellous bone graft from the distal tibia for reconstructive surgery of the foot and ankle. J Foot Ankle Surg..

[19] Friedlaender G.E. (1982). Bone-banking. J. Bone Jt. Surg. Am..

[20] Migliorini F., Eschweiler J., Maffulli N., Schenker H., Driessen A., Rath B., Tingart M. (2021). Autologous Matrix Induced Chondrogenesis (AMIC) Compared to Microfractures for Chondral Defects of the Talar Shoulder: A Five-Year Follow-Up Prospective Cohort Study. Life.

[21] Migliorini F., Eschweiler J., Maffulli N., Schenker H., Baroncini A., Tingart M., Rath B. (2021). Autologous Matrix-Induced Chondrogenesis (AMIC) and Microfractures for Focal Chondral Defects of the Knee: A Medium-Term Comparative Study. Life.

[22] Migliorini F., Eschweiler J., Maffulli N., Driessen A., Rath B., Tingart M., Schenker H. (2021). Management of Patellar Chondral Defects with Autologous Matrix Induced Chondrogenesis (AMIC) Compared to Microfractures: A Four Years Follow-Up Clinical Trial. Life.

[23] Finkemeier C.G. (2002). Bone-grafting and bone-graft substitutes. J. Bone Jt. Surg. Am..

[24] Gazdag A.R., Lane J.M., Glaser D., Forster R.A. (1995). Alternatives to Autogenous Bone Graft: Efficacy and Indications. J. Am. Acad. Orthop. Surg..

[25] Dimitriou R., Jones E., McGonagle D., Giannoudis P.V. (2011). Bone regeneration: Current concepts and future directions. BMC Med..

[26] Asmus A., Vogel K., Vogel A., Eichenauer F., Kim S., Eisenschenk A. (2020). Pedicled vascularized iliac bone graft for treatment of osteonecrosis of the femoral head. Oper. Orthop. Traumatol..

[27] Ghassemi A., Ghassemi M., Riediger D., Hilgers R.D., Gerressen M. (2009). Comparison of donor-site engraftment after harvesting vascularized and nonvascularized iliac bone grafts. J. Oral Maxillofac. Surg..

[28] Dell P.C., Burchardt H., Glowczewskie F.P. (1985). A roentgenographic, biomechanical, and histological evaluation of vascularized and non-vascularized segmental fibular canine autografts. J. Bone Jt. Surg. Am..

[29] El-Alfy B., Abulsaad M., Abdelnaby W.L. (2018). The use of free nonvascularized fibular graft in the induced membrane technique to manage post-traumatic bone defects. Eur. J. Orthop. Surg. Traumatol..

[30] Marechek A., AlShare A., Pack S., Demko C., Quereshy F.A., Baur D. (2019). Nonvascularized Bone Grafts for Reconstruction of Segmental Mandibular Defects: Is Length of Graft a Factor of Success?. J. Oral Maxillofac. Surg..

[31] Allsopp B.J., Hunter-Smith D.J., Rozen W.M. (2016). Vascularized versus Nonvascularized Bone Grafts: What Is the Evidence?. Clin. Orthop. Relat. Res..

[32] Madison R.D., Nowotarski P.J. (2019). The Reamer-Irrigator-Aspirator in Nonunion Surgery. Orthop. Clin. N. Am..

[33] Meeder P.J., Eggers C. (1994). Techniques for obtaining autogenous bone graft. Injury.

[34] Judet H., Gilbert A., Mathoulin C., Judet J., Judet T., Siguier M., Brumpt B. (1991). Reconstruction of loss of bony substance in limbs by free vascularized fibula transplant. Chirurgie.

[35] Osborn T.M., Helal D., Mehra P. (2018). Iliac crest bone grafting for mandibular reconstruction: 10-year experience outcomes. J. Oral Biol. Craniofac. Res..

[36] Salawu O.N., Babalola O.M., Ahmed B.A., Ibraheem G.H., Kadir D.M. (2017). Comparative Study of Proximal Tibia and Iliac Crest Bone Graft Donor Sites in Treatment of Orthopaedic Pathologies. Malays. Orthop. J..

[37] Gotze C., Nieder C., Felder H., Peterlein C.D., Migliorini F. (2021). AMIC for traumatic focal osteochondral defect of the talar shoulder: A 5 years follow-up prospective cohort study. BMC Musculoskelet. Disord..

[38] Gotze C., Nieder C., Felder H., Migliorini F. (2020). AMIC for Focal Osteochondral Defect of the Talar Shoulder. Life.

[39] Migliorini F., Maffulli N., Baroncini A., Knobe M., Tingart M., Eschweiler J. (2021). Matrix-induced autologous chondrocyte implantation versus autologous matrix-induced chondrogenesis for chondral defects of the talus: A systematic review. Br. Med. Bull..

[40] Migliorini F., Maffulli N., Baroncini A., Eschweiler J., Tingart M., Betsch M. (2021). Failure and progression to total hip arthroplasty among the treatments for femoral head osteonecrosis: A Bayesian network meta-analysis. Br. Med. Bull..

[41] Schnee C.L., Freese A., Weil R.J., Marcotte P.J. (1997). Analysis of harvest morbidity and radiographic outcome using autograft for anterior cervical fusion. Spine.

[42] Merritt A.L., Spinnicke A., Pettigrew K., Alamin T.F. (2010). Gluteal-sparing approach for posterior iliac crest bone graft: Description of a new technique and assessment of morbidity in ninety-two patients after spinal fusion. Spine.

[43] Pollock R., Alcelik I., Bhatia C., Chuter G., Lingutla K., Budithi C., Krishna M. (2008). Donor site morbidity following iliac crest bone harvesting for cervical fusion: A comparison between minimally invasive and open techniques. Eur. Spine J..

[44] Robertson P.A., Wray A.C. (2001). Natural history of posterior iliac crest bone graft donation for spinal surgery: A prospective analysis of morbidity. Spine.

[45] Schwartz C.E., Martha J.F., Kowalski P., Wang D.A., Bode R., Li L., Kim D.H. (2009). Prospective evaluation of chronic pain associated with posterior autologous iliac crest bone graft harvest and its effect on postoperative outcome. Health Qual. Life Outcomes.

[46] Boucree T., McLaughlin D., Akrawe S., Darian V., Siddiqui A. (2017). Posterior Iliac Crest Bone Graft: How Much Is Enough?. J. Craniofac. Surg..

[47] Hu R., Hearn T., Yang J. (1995). Bone graft harvest site as a determinant of iliac crest strength. Clin. Orthop. Relat. Res..

[48] Gil-Albarova J., Gil-Albarova R. (2012). Donor site reconstruction in iliac crest tricortical bone graft: Surgical technique. Injury.

[49] Brawley S.C., Simpson R.B. (2006). Results of an alternative autogenous iliac crest bone graft harvest method. Orthopedics.

[50] Bimmel R., Govaers K. (2006). Does harvesting of iliac bone grafts with an acetabular reamer reduce complication rate?. Acta Orthop. Belg..

[51] Pape H.C., Evans A., Kobbe P. (2010). Autologous bone graft: Properties and techniques. J. Orthop. Trauma.

[52] Ebraheim N.A., Elgafy H., Xu R. (2001). Bone-graft harvesting from iliac and fibular donor sites: Techniques and complications. J. Am. Acad. Orthop. Surg..

[53] Ilankovan V., Stronczek M., Telfer M., Peterson L.J., Stassen L.F., Ward-Booth P. (1998). A prospective study of trephined bone grafts of the tibial shaft and iliac crest. Br. J. Oral Maxillofac. Surg..

[54] Behairy Y.M., Al-Sebai W. (2001). A modified technique for harvesting full-thickness iliac crest bone graft. Spine.

[55] Tashjian R.Z., Horwitz D.S. (2009). Healing and graft-site morbidity rates for midshaft clavicle nonunions treated with open reduction and internal fixation augmented with iliac crest aspiration. Am. J. Orthop. (Belle Mead NJ).

[56] Westrich G.H., Geller D.S., O’Malley M.J., Deland J.T., Helfet D.L. (2001). Anterior iliac crest bone graft harvesting using the corticocancellous reamer system. J. Orthop. Trauma.

[57] Belthur M.V., Conway J.D., Jindal G., Ranade A., Herzenberg J.E. (2008). Bone graft harvest using a new intramedullary system. Clin. Orthop. Relat. Res..

[58] Freije M.R. (2003). Pure + easy. Selecting a domestic water disinfection system. Health Facil. Manag..

[59] Catanzariti A., Karlock L. (1996). The application of allograft bone in foot and ankle surgery. J. Foot Ankle Surg..

[60] Laursen M., Christensen F.B., Bunger C., Lind M. (2003). Optimal handling of fresh cancellous bone graft: Different peroperative storing techniques evaluated by in vitro osteoblast-like cell metabolism. Acta. Orthop. Scand..

[61] Lindsey R.W., Probe R., Miclau T., Alexander J.W., Perren S.M. (1993). The effects of antibiotic-impregnated autogeneic cancellous bone graft on bone healing. Clin. Orthop. Relat. Res..

[62] Maus U., Andereya S., Gravius S., Siebert C.H., Schippmann T., Ohnsorge J.A., Niedhart C. (2008). How to store autologous bone graft perioperatively: An in vitro study. Arch. Orthop. Trauma Surg..

[63] Chan Y.S., Ueng S.W., Wang C.J., Lee S.S., Chen C.Y., Shin C.H. (2000). Antibiotic-impregnated autogenic cancellous bone grafting is an effective and safe method for the management of small infected tibial defects: A comparison study. J. Trauma.

[64] Wessel A.R., Crist B.D., Stannard J.P., Della Rocca G.J., Stoker A.M., Bozynski C.C., Cook C.R., Kuroki K., Ahner C.E., Cook J.L. (2018). Assessment of Reamer Irrigator Aspirator System (RIA) filtrate for its osteoinductive potential in a validated animal model. Injury.

[65] Herford A.S., King B.J., Audia F., Becktor J. (2003). Medial approach for tibial bone graft: Anatomic study and clinical technique. J. Oral Maxillofac. Surg..

[66] Boone D.W. (2003). Complications of iliac crest graft and bone grafting alternatives in foot and ankle surgery. Foot Ankle Clin..

[67] O’Keeffe R.M., Riemer B.L., Butterfield S.L. (1991). Harvesting of autogenous cancellous bone graft from the proximal tibial metaphysis. A review of 230 cases. J. Orthop. Trauma.

[68] Alt V., Nawab A., Seligson D. (1999). Bone grafting from the proximal tibia. J. Trauma.

[69] Wang K., Almeida L.E., Olsson A.B. (2007). Volume analysis of the proximal tibial metaphysis. J. Oral Maxillofac. Surg..

[70] Geideman W., Early J.S., Brodsky J. (2004). Clinical results of harvesting autogenous cancellous graft from the ipsilateral proximal tibia for use in foot and ankle surgery. Foot Ankle Int..

[71] Becker S.T., Warnke P.H., Behrens E., Wiltfang J. (2011). Morbidity after iliac crest bone graft harvesting over an anterior versus posterior approach. J. Oral Maxillofac. Surg..

[72] Biddulph S.L. (1999). Bone donor site. Iliac crest or distal radius?. J. Hand Surg. Br..

[73] Daigre J.L., DeMill S.L., Hyer C.F. (2016). Assessment of Bone Marrow Aspiration Site Pain in Foot and Ankle Surgery. Foot Ankle Spec..

[74] Lindberg E.J., Katchis S.D., Smith R.W. (1996). Quantitative analysis of cancellous bone graft available from the greater trochanter. Foot Ankle Int..

[75] Hayes W.R., Smith R.W. (1996). Trochanteric bone grafts in foot and ankle surgery. Foot Ankle Int..

[76] Patel J.C., Watson K., Joseph E., Garcia J., Wollstein R. (2003). Long-term complications of distal radius bone grafts. J. Hand Surg. Am..

[77] Bruno R.J., Cohen M.S., Berzins A., Sumner D.R. (2001). Bone graft harvesting from the distal radius, olecranon, and iliac crest: A quantitative analysis. J. Hand Surg. Am..

[78] Eglseder W.A., Elliott M.J. (2002). Nonunions of the distal radius. Am. J. Orthop. (Belle Mead NJ).

[79] Kovar F.M., Wozasek G.E. (2011). Bone graft harvesting using the RIA (reaming irrigation aspirator) system—A quantitative assessment. Wien. Klin. Wochenschr..

[80] Han F., Peter L., Lau E.T., Thambiah J., Murphy D., Kagda F.H. (2015). Reamer Irrigator Aspirator bone graft harvesting: Complications and outcomes in an Asian population. Injury.

[81] Haubruck P., Ober J., Heller R., Miska M., Schmidmaier G., Tanner M.C. (2018). Complications and risk management in the use of the reaming-irrigator-aspirator (RIA) system: RIA is a safe and reliable method in harvesting autologous bone graft. PLoS ONE.

[82] Silber J.S., Anderson D.G., Daffner S.D., Brislin B.T., Leland J.M., Hilibrand A.S., Vaccaro A.R., Albert T.J. (2003). Donor site morbidity after anterior iliac crest bone harvest for single-level anterior cervical discectomy and fusion. Spine.

[83] Fernyhough J.C., Schimandle J.J., Weigel M.C., Edwards C.C., Levine A.M. (1992). Chronic donor site pain complicating bone graft harvesting from the posterior iliac crest for spinal fusion. Spine.

[84] Boehm K.S., Al-Taha M., Morzycki A., Samargandi O.A., Al-Youha S., LeBlanc M.R. (2019). Donor Site Morbidities of Iliac Crest Bone Graft in Craniofacial Surgery: A Systematic Review. Ann. Plast. Surg..

[85] Lementowski P.W., Lucas P., Taddonio R.F. (2010). Acute and chronic complications of intracortical iliac crest bone grafting versus the traditional corticocancellous technique for spinal fusion surgery. Orthopedics.

[86] Babbi L., Barbanti-Brodano G., Gasbarrini A., Boriani S. (2016). Iliac crest bone graft: A 23-years hystory of infection at donor site in vertebral arthrodesis and a review of current bone substitutes. Eur. Rev. Med. Pharmacol. Sci..

[87] Armaghani S.J., Even J.L., Zern E.K., Braly B.A., Kang J.D., Devin C.J. (2016). The Evaluation of Donor Site Pain after Harvest of Tricortical Anterior Iliac Crest Bone Graft for Spinal Surgery: A Prospective Study. Spine.

[88] Arrington E.D., Smith W.J., Chambers H.G., Bucknell A.L., Davino N.A. (1996). Complications of iliac crest bone graft harvesting. Clin. Orthop. Relat. Res..

[89] Banwart J.C., Asher M.A., Hassanein R.S. (1995). Iliac crest bone graft harvest donor site morbidity. A statistical evaluation. Spine.

[90] Hofmann A., Gorbulev S., Guehring T., Schulz A.P., Schupfner R., Raschke M., Huber-Wagner S., Rommens P.M., Group C.E.S. (2020). Autologous Iliac Bone Graft Compared with Biphasic Hydroxyapatite and Calcium Sulfate Cement for the Treatment of Bone Defects in Tibial Plateau Fractures: A Prospective, Randomized, Open-Label, Multicenter Study. J. Bone Jt. Surg. Am..

[91] DePalma A.F., Rothman R.H., Lewinnek G.E., Canale S.T. (1972). Anterior interbody fusion for severe cervical disc degeneration. Surg. Gynecol. Obstet..

[92] Canady J.W., Zeitler D.P., Thompson S.A., Nicholas C.D. (1993). Suitability of the iliac crest as a site for harvest of autogenous bone grafts. Cleft Palate Craniofac. J..

[93] Sheha E.D., Meredith D.S., Shifflett G.D., Bjerke B.T., Iyer S., Shue J., Nguyen J., Huang R.C. (2018). Postoperative pain following posterior iliac crest bone graft harvesting in spine surgery: A prospective, randomized trial. Spine J..

[94] DeOrio J.K., Farber D.C. (2005). Morbidity associated with anterior iliac crest bone grafting in foot and ankle surgery. Foot Ankle Int..

[95] Huang Y.C., Chen C.Y., Lin K.C., Renn J.H., Tarng Y.W., Hsu C.J., Chang W.N., Yang S.W. (2018). Comparing morbidities of bone graft harvesting from the anterior iliac crest and proximal tibia: A retrospective study. J. Orthop. Surg. Res..

[96] Schizas C., Triantafyllopoulos D., Kosmopoulos V., Stafylas K. (2009). Impact of iliac crest bone graft harvesting on fusion rates and postoperative pain during instrumented posterolateral lumbar fusion. Int. Orthop..

[97] Haws B.E., Khechen B., Patel D.V., Yoo J.S., Guntin J.A., Cardinal K.L., Ahn J., Singh K. (2019). Impact of Iliac Crest Bone Grafting on Postoperative Outcomes and Complication Rates Following Minimally Invasive Transforaminal Lumbar Interbody Fusion. Neurospine.

[98] Conway J.D. (2010). Autograft and nonunions: Morbidity with intramedullary bone graft versus iliac crest bone graft. Orthop. Clin. N. Am..

[99] Cox G., Jones E., McGonagle D., Giannoudis P.V. (2011). Reamer-irrigator-aspirator indications and clinical results: A systematic review. Int. Orthop..

[100] Jakma T.S., Roling M.A., Punt B., Reynders-Frederix P. (2014). More adverse events than expected in the outcome after use of the reamer-irrigator-aspirator. Eur. J. Trauma Emerg. Surg..

[101] Taylor B.C., Triplet J.J., Johnson D.B., Sharpe B.D., Sullivan B., Canini C. (2018). Retrograde Femoral Bone Graft Acquisition Using the Reamer-Irrigator-Aspirator. J. Long Term Eff. Med. Implants.

[102] Stafford P.R., Norris B.L. (2010). Reamer-irrigator-aspirator bone graft and bi Masquelet technique for segmental bone defect nonunions: A review of 25 cases. Injury.

[103] Kanakaris N.K., Morell D., Gudipati S., Britten S., Giannoudis P.V. (2011). Reaming Irrigator Aspirator system: Early experience of its multipurpose use. Injury.

[104] Burstein F.D., Simms C., Cohen S.R., Work F., Paschal M. (2000). Iliac crest bone graft harvesting techniques: A comparison. Plast. Reconstr. Surg..

[105] Delawi D., Dhert W.J., Castelein R.M., Verbout A.J., Oner F.C. (2007). The incidence of donor site pain after bone graft harvesting from the posterior iliac crest may be overestimated: A study on spine fracture patients. Spine.

[106] Metsemakers W.J., Claes G., Terryn P.J., Belmans A., Hoekstra H., Nijs S. (2019). Reamer-Irrigator-Aspirator bone graft harvesting for treatment of segmental bone loss: Analysis of defect volume as independent risk factor for failure. Eur. J. Trauma Emerg. Surg..

[107] Quintero A.J., Tarkin I.S., Pape H.C. (2010). Technical tricks when using the reamer irrigator aspirator technique for autologous bone graft harvesting. J. Orthop. Trauma.

[108] Almaiman M., Al-Bargi H.H., Manson P. (2013). Complication of anterior iliac bone graft harvesting in 372 adult patients from may 2006 to may 2011 and a literature review. Craniomaxillofac. Trauma Reconstr..

[109] Lowe J.A., Crist B.D., Pfeiffer F., Carson W.L. (2015). Predicting Reduction in Torsional Strength by Concentric/Eccentric RIA Reaming Normal and Osteoporotic Long Bones (Femurs). J. Orthop. Trauma.

